# Analysis of Factors Related to Early Left Ventricular Dysfunction in Hypertensive Patients with Preserved Ejection Fraction Using Speckle Tracking Echocardiography: A Cross-Sectional Study in Vietnam

**DOI:** 10.3390/diagnostics15020222

**Published:** 2025-01-19

**Authors:** Hoang M. Tran, Hung P. Truong, Cuong C. Tran, Tuan M. Vo, Dung N. Q. Nguyen, Liem T. Dao

**Affiliations:** 1 Medicine Faculty, University of Medicine and Pharmacy at Ho Chi Minh City, Ho Chi Minh City 7000, Vietnam; tranminhhoang@ump.edu.vn (H.M.T.); truongphihung2007@ump.edu.vn (H.P.T.); dr.trancaocuong@ump.edu.vn (C.C.T.); 2 Medicine Faculty, University of Health Science, Vietnam National University, Ho Chi Minh City 7000, Vietnam; nnqdung@medvnu.edu.vn (D.N.Q.N.); dtliem@medvnu.edu.vn (L.T.D.)

**Keywords:** echocardiography, hypertension, left ventricular dysfunction, preserved ejection fraction, Vietnam

## Abstract

**Objective:** The purpose of this research was to assess the factors linked to early left ventricular (LV) dysfunction in hypertensive patients who have preserved ejection fraction (EF ≥ 50%) using speckle tracking echocardiography. **Methods**: A cross-sectional study was carried out involving 579 outpatients recruited from City International Hospital in Ho Chi Minh City using a total sampling approach, where echocardiographic measurements and clinical data were gathered and analyzed. **Results:** The prevalence of LV diastolic dysfunction among hypertensive individuals was found to be 45.25%, with 9.15% showing abnormal global longitudinal strain (GLS). Factors such as being over the age of 60, having diabetes, concentric LV hypertrophy, concentric LV remodeling, and LV diastolic dysfunction were identified as correlating with abnormal GLS (*p* < 0.05). In contrast, other cardiovascular risk factors, including smoking and dyslipidemia, did not significantly influence the GLS index (*p* > 0.05). **Conclusions:** Key factors including diabetes, diastolic dysfunction, concentric hypertrophy, and concentric remodeling of the LV are significant predictors of abnormal GLS. These results are important for the management of hypertensive patients aimed at enhancing cardiac function.

## 1. Introduction

Hypertension has emerged as a significant public health concern worldwide. Current estimates suggest that approximately 30–45% of the general population suffer from hypertension, with prevalence increasing with age [1]. This condition results in complications affecting essential organs, including the heart, brain, eyes, kidneys, and peripheral arteries [2]. Hypertensive cardiomyopathy encompasses various cardiac issues stemming from hypertension, such as left ventricular (LV) hypertrophy, and both systolic and diastolic dysfunction. Clinical signs of hypertensive cardiomyopathy may include arrhythmia and heart failure. GLS enables early detection of dysfunction by highlighting connections between systolic and diastolic left ventricle function. Myocardial deformation, as evaluated by GLS, and traditional hemodynamic parameters share a common basis, which underpins the benefits of assessing myocardial deformation in patients with preserved ejection fraction. The occurrence of myocardial hypertrophy linked to hypertension depends on blood pressure levels, with around 20% affected by mild hypertension and a rate of nearly 100% among those with severe hypertension or related complications [3]. The Framingham study revealed that the likelihood of developing heart failure relies on the geometric configuration of the left ventricle: concentric hypertrophy is often associated with heart failure with preserved ejection fraction, while eccentric hypertrophy can lead to heart failure with reduced ejection fraction over a 21-year period [4]. In the hypertensive population, only 24% show eccentric hypertrophy, while 40% have concentric hypertrophy, indicating that some hypertensive individuals may experience heart failure without showing signs of myocardial hypertrophy [5]. LV diastolic dysfunction plays a critical role in hypertensive cardiomyopathy and can progress to symptomatic congestive heart failure, affecting about 40% of hypertensive patients. It is characterized by a thickened left ventricular wall, consistently elevating end-diastolic pressure, and enlarged left atrial volume [6]. Conversely, LV systolic dysfunction is relatively rare in cases of hypertensive cardiomyopathy, occurring in roughly 3–6% of hypertensive individuals, with eccentric hypertrophy posing a considerable risk for its development. If left untreated, LV remodeling and hypertrophy can result in ventricular dilation and subsequent heart failure [7]. Consequently, early identification of cardiac dysfunction is crucial for clinicians to formulate improved treatment approaches.

In clinical settings, echocardiography is frequently the preferred diagnostic instrument. M-mode and Simpson’s method are typically employed to assess left ventricular (LV) functionality; however, these approaches are semi-quantitative and subjective when it comes to measuring intimal thickness. They tend to identify cardiac issues only after complications such as LV hypertrophy or chamber enlargement have developed, which leads to relatively low sensitivity in recognizing subtle anomalies in cardiac function.

Recently, cardiac strain indices have been utilized to identify impairments in cardiac function early on, even prior to the onset of LV hypertrophy. Numerous studies indicate that speckle tracking echocardiography can detect slight heart function impairments in cases of minor myocardial dysfunction, even in the absence of structural changes in the heart [8,9]. Furthermore, speckle tracking echocardiography assesses both systolic and diastolic functions while also evaluating myocardial fibrosis, aiding in differentiating between hypertensive-induced left ventricular hypertrophy and that resulting from other etiologies [10]. Additionally, this technique allows for the evaluation of heart function from various perspectives, irrespective of imaging angles [11,12].

The goal of this study was to analyze the factors influencing early LV dysfunction in hypertensive patients who maintain a preserved ejection fraction (≥50%) using tissue tracking echocardiography.

## 2. Research Methods

### 2.1. Study Population

A cross-sectional study involving 579 hypertensive outpatients was recruited from City International Hospital in Ho Chi Minh City, Vietnam, using a total sampling approach from October 2020 to September 2023. Participants were included if they were aged 40 or older, had a systolic blood pressure of 140 mmHg or higher, a diastolic blood pressure of 90 mmHg or higher, or were receiving treatment for hypertension at least twice, with an ejection fraction of 50% or greater assessed using the 2D Simpson method. Patients were excluded from the study if they had secondary hypertension, cardiomyopathy, moderate to severe valvular disease, ischemic heart disease, or arrhythmia.

Data on clinical characteristics such as height, weight, body mass index (BMI), duration of hypertension, and cardiovascular risk factors (including diabetes, dyslipidemia, and smoking) were obtained from the hospital management system. All participants provided informed consent, and the study received approval from the ethics committee.

### 2.2. Echocardiographic Assessment

Standard and two-dimensional speckle tracking echocardiography were conducted using GE Vivid TM T8 machines, (GE HealthCare, Chicago, IL, USA) capturing images during end-expiration while the patient was positioned in the left lateral decubitus posture. Echocardiographic images were recorded at rates exceeding 55 frames per second, with the procedure carried out by 2 physicians, each possessing over 5 years of experience. In cases of result discrepancies, the average of both assessments was utilized.

**The assessment of left ventricular diastolic dysfunction followed the 2016 recommendations from ASE/EACVI** [13]:

- An E/A (early to late mitral filling velocity ratio) of ≤0.8 and E ≤ 50 cm/s indicates grade I left ventricular diastolic dysfunction, characterized by normal left atrial or left ventricular filling pressures.
- If E/A is ≤0.8 with E > 50 cm/s, or if 0.8 < E/A < 2, further evaluation is needed based on three additional criteria: (1) an average E/e’ ratio (average mitral-to-peak early diastolic annular) exceeding 14; (2) a tricuspid regurgitation velocity greater than 2.8 m/s; and (3) a left atrial volume index exceeding 34 mL/m^2^. If fewer than two criteria are satisfied, it is classified as grade I dysfunction with normal filling pressures. Meeting at least two criteria indicate grade II diastolic dysfunction with elevated filling pressures. In the case of only two criteria being available: (1) the absence of both criteria leads to grade I classification with normal pressures; (2) meeting one criterion means that left atrial or ventricular filling pressure cannot be determined, nor can the dysfunction grade; (3) meeting both criteria suggests grade II diastolic dysfunction with elevated pressures.
- An E/A of ≥2 indicates grade III diastolic dysfunction with elevated left atrial or ventricular filling pressures.

**Left Ventricular Geometry is assessed following the ASE/EACVI 2016 guidelines** [14].

- Normal cardiac geometry is recognized when the left ventricular mass index (LVMI) is at or below 115 g/m^2^ for males and 95 g/m^2^ for females, along with a relative wall thickness (RWT) of 0.42 or less.
- Concentric hypertrophy occurs when LVMI exceeds 115 g/m^2^ in males or 95 g/m^2^ in females and RWT is greater than 0.42.
- Eccentric hypertrophy is noted when LVMI is above 115 g/m^2^ for males or 95 g/m^2^ for females, while RWT remains at or below 0.42.
- Concentric remodeling is characterized by an LVMI of 115 g/m^2^ or less in males and 95 g/m^2^ or less in females, accompanied by an RWT that exceeds 0.42.

**Figure 1:**
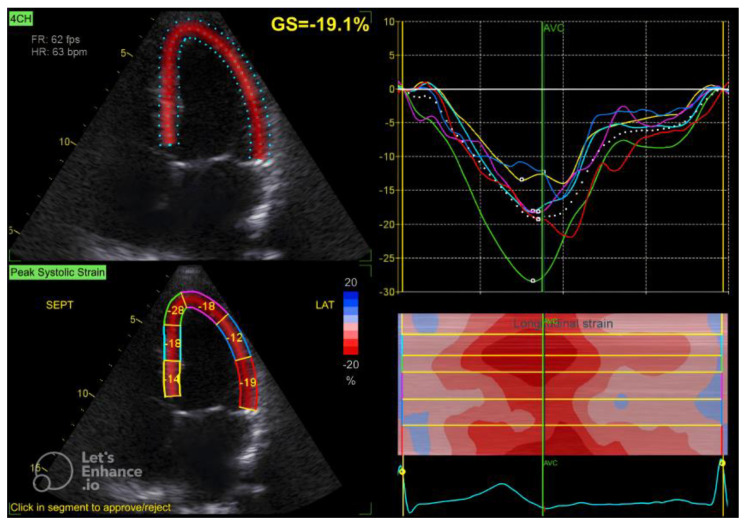
Four-chamber apical view in speckle tracking.

Figure 1: Ejection fraction with LV mass index and RWT in a normal 49-year-old woman.

**Global Longitudinal Strain (GLS) Measurement**

**Figure 2:**
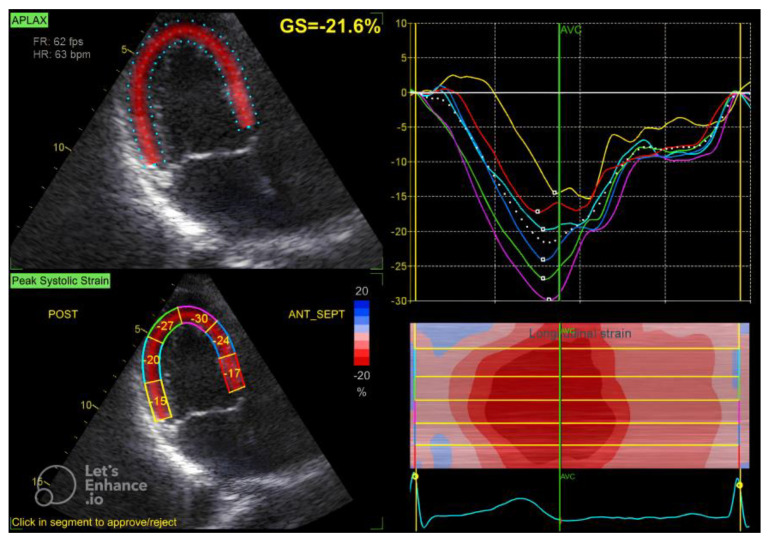
Three-chamber apical view in speckle tracking.

**Figure 3:**
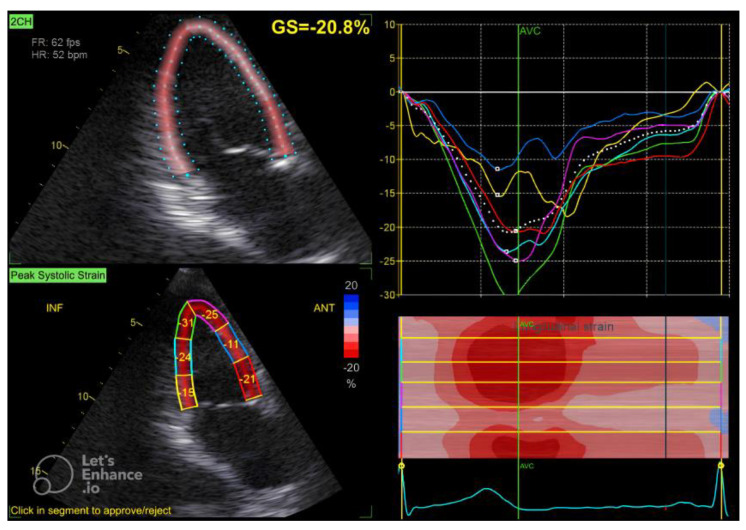
Two-chamber apical view in speckle tracking.

The GLS value of the left ventricle is determined by averaging the regional myocardial strain from three different views: the apical 4-chamber (Figure 1), 3-chamber (Figure 2), and 2-chamber (Figure 3) perspectives. The selection of images and the tracing of the endocardial borders were conducted following the 2015 ASE/EACVI guidelines [15]. Speckle-tracking echocardiography images were analyzed using AFI 3.0 software available from GE. An abnormal GLS is identified as a value that exceeds −16.7% [16].

### 2.3. Statistical Analysis

Statistical analysis was performed using Stata version 18. The Kolmogorov–Smirnov test was utilized to assess the normality of continuous variables, which were reported as mean ± standard deviation or median, while categorical variables were expressed as frequencies and percentages. *t*-tests and ANOVA were employed for comparing groups with continuous variables that followed a normal distribution. For continuous variables that were not normally distributed, the Mann–Whitney U test and Kruskal–Wallis’s test were used; the Chi-squared test was applied for categorical variables. To determine the predictors of GLS decline and LV diastolic dysfunction, we implemented the lasso technique alongside evaluations using AIC, BIC, and assessed multicollinearity through VIF for selecting the multivariable logistic regression model.

## 3. Results

### 3.1. Population Characteristics

Out of the total 579 patients, 61.49% (*n* = 356) were female and 38.51% (*n* = 223) were male. The average age of participants was 64.04 ± 10.74 years, with 67.01% being 60 years or older. In terms of body mass index (BMI), 41.28% were classified as obese, while 29.02% were categorized as overweight. The rates of diabetes and dyslipidemia among the population were found to be 24.01% and 16.58%, respectively. For further details on the demographic and clinical features of the subjects, please refer to Table 1.

### 3.2. Echocardiographic Function and GLS Index

Table 2 presents the echocardiographic features of all participants. The mean global longitudinal strain (GLS) for the study cohort was −20.25 ± 2.53. Diastolic dysfunction was identified in 45.25% of the subjects, while left ventricular hypertrophy was observed in 19.34% of the individuals. Abnormal GLS, defined as GLS greater than −16.7%, was found in 9.15% of the patients.

### 3.3. Association Between Cardiovascular Risk Factors and Echocardiographic Indices

Table 3, Table 4, Table 5, Table 6 and Table 7 present the correlation between cardiovascular risk factors and echocardiographic assessments. Diastolic function indices showed a relationship with age, whereas BMI, smoking habits, and dyslipidemia were related to LV mass index (LVMI) and global longitudinal strain (GLS). Additionally, diabetes mellitus was found to correlate with diastolic function, left ventricular hypertrophy, and GLS.

### 3.4. Risk Factors Associated with LV Dysfunction in Hypertensive Patients with Preserved EF

Factors such as age over 60, diabetes mellitus, uncontrolled blood were associated with LV diastolic dysfunction in hypertensive patients (Table 8).

In the analysis of hypertensive patients, those displaying reduced global longitudinal strain (GLS) exhibited a notably greater incidence of diabetes, dyslipidemia, left ventricular (LV) diastolic dysfunction, and LV remodeling in comparison to individuals with normal GLS. Additionally, within the reduced GLS cohort, there was a higher proportion of patients aged 60 and older who had uncontrolled blood pressure compared to the normal GLS group (Table 9).

### 3.5. Multivariate Analysis

Individuals aged 60 years and above (OR = 1.70, 95% CI 1.29–2.12, *p* < 0.001), those with uncontrolled hypertension (OR = 1.92, 95% CI 1.77–2.85, *p* < 0.001), patients with diabetes mellitus (OR = 1.54, 95% CI 1.02–2.33, *p* = 0.041), and individuals who are obese (OR = 1.73, 95% CI 1.11–2.70, *p* = 0.016) are recognized as significant independent risk factors for impaired left ventricular diastolic function in patients with hypertension (refer to Table 10).

The research found that individuals aged 60 and older had an odds ratio (OR) of 2.32 (95% CI 1.67–4.88, *p* = 0.020). Additionally, uncontrolled blood pressure was associated with an OR of 2.02 (95% CI 1.75–3.99, *p* = 0.031). The presence of diabetes mellitus corresponded to an OR of 2.06 (95% CI 1.09–3.88, *p* = 0.026). Furthermore, left ventricular (LV) concentric hypertrophy was linked to a significant OR of 5.78 (95% CI 1.75–19.12, *p* = 0.004), while LV remodeling had an OR of 4.82 (95% CI 1.34–17.34, *p* = 0.016). Lastly, LV diastolic dysfunction was associated with an OR of 3.51 (95% CI 1.74–7.09, *p* < 0.001), all of which were factors related to a reduced global longitudinal strain (GLS), as outlined in Table 11.

## 4. Discussion

This research investigated elements associated with the onset of left ventricular dysfunction in hypertensive individuals who maintain preserved ejection fraction. The findings highlight several important aspects concerning the association between cardiovascular risk factors and the global longitudinal strain (GLS) index, along with left ventricular function.

Relationship between age and abnormal GLS: The study findings suggest that age significantly affects GLS reduction. Individuals aged 60 and above exhibit a notably higher incidence of GLS impairment, which may result from the natural aging changes in the cardiovascular system, such as myocardial fibrosis and decreased elasticity of blood vessel walls. This aligns with earlier research indicating that GLS generally declines as age increases, especially among those over 60 [17,18]. Nonetheless, certain studies have reported no correlation between age and GLS [19,20]. This variability can be attributed to the notion that age impacts GLS reduction only in patients with cardiovascular conditions, while it does not influence healthy individuals. Research conducted by Potter et al. involving participants aged 65 and older revealed an increase in GLS as age progressed, along with higher NT-proBNP levels [21]. Timely identification of left ventricular dysfunction in this demographic is essential for prompt intervention, which can help mitigate the risk of heart failure development.

Impact of diabetes on LV function and GLS: Diabetes is recognized as a considerable contributor to abnormal global longitudinal strain (GLS). Research indicates that individuals with diabetes face an elevated risk of left ventricular dysfunction, which may stem from microvascular injury, angiogenesis, and myocardial fibrosis associated with the condition. These observations align with earlier research that identifies diabetes as an independent risk factor for diabetic cardiomyopathy, leading to diminished myocardial contractile capacity even prior to the manifestation of clinical symptoms, particularly in those with hypertension [22,23,24]. The investigation conducted by Nakai et al. revealed that GLS in diabetic patients was significantly lower when compared to healthy individuals aged 25 years. Additionally, 43% of the diabetic cohort demonstrated a decreased GLS (cut-off value of −17.2) while still maintaining preserved left ventricular ejection fraction (LVEF) [25]. Multiple studies have also indicated that LV GLS should be regarded as the principal marker for evaluating cardiac dysfunction associated with diabetes in patients who have preserved LVEF, as opposed to focusing solely on diastolic dysfunction of the left ventricle [26,27,28].

Role of diastolic dysfunction and LV structural remodeling: The findings suggest a strong correlation between diastolic dysfunction and left ventricular (LV) structural changes, such as concentric hypertrophy and concentric remodeling, with abnormal global longitudinal strain (GLS). Diastolic dysfunction serves as an early indicator of LV impairment in hypertensive individuals, especially when left ventricular ejection fraction (LVEF) remains normal [29,30]. These structural changes in the LV indicate that increased afterload is placed on the heart, resulting in greater resistance to myocardial contraction, which ultimately compromises left ventricular performance [29]. The Framingham study indicated that the likelihood of developing heart failure is influenced by the geometric configurations of the left ventricle; concentric hypertrophy often progresses to heart failure with preserved ejection fraction, while eccentric hypertrophy is more likely to lead to heart failure with reduced ejection fraction over a 21-year period [4]. These results carry significant clinical relevance, underscoring the necessity for timely assessment and intervention concerning diastolic dysfunction and LV structural alterations to avert the advancement toward heart failure.

Uncontrolled hypertension and GLS impairment: Unmanaged hypertension serves as an autonomous risk factor linked to abnormal global longitudinal strain (GLS), highlighting the essential need for blood pressure management to avert cardiac impairment. Patients with uncontrolled hypertension face a significant risk of GLS deterioration, suggesting that persistent high blood pressure may result in cardiovascular harm due to fibrosis and left ventricular hypertrophy [31,32]. Research conducted by Bendiab et al. revealed that unmanaged hypertension is a standalone risk factor contributing to diminished GLS in hypertensive individuals with preserved left ventricular ejection fraction (LVEF) [33]. This finding further emphasizes the necessity of rigorous blood pressure regulation in patients to enhance cardiac function and lessen the likelihood of cardiovascular issues.

Role of a myocardial strain index (GLS) in early detection of cardiac dysfunction: Utilizing global longitudinal strain (GLS) to identify early cardiac dysfunction in hypertensive patients with normal ejection fraction is an emerging and promising strategy. GLS can reveal declines in contractile function sooner than other measurements, such as ejection fraction (EF), particularly in the absence of structural alterations like left ventricular hypertrophy or chamber enlargement [34,35]. These research findings emphasize that GLS serves as a valuable instrument for tracking the progression of cardiovascular disease in this specific patient population. In our investigation, 16 out of 53 patients (30.19%) demonstrated decreased GLS without accompanying diastolic dysfunction of the left ventricle (LV). This indicates that diminished GLS may occur alongside LV dysfunction in hypertensive individuals with preserved left ventricular ejection fraction (LVEF), suggesting that GLS might be a more sensitive indicator of predicting LV dysfunction in this demographic.

### Study Limitations and Future Research Directions

This research has various limitations, notably its cross-sectional design, which does not allow for the establishment of causal links between risk factors and GLS impairment. Additionally, variables such as the length of hypertension and the management of related conditions may influence outcomes but have not been adequately addressed. Consequently, there is a need for cohort studies to validate these risk factors over time.

## 5. Conclusions

The research highlights important risk factors associated with left ventricular dysfunction and GLS in hypertensive individuals who maintain preserved ejection fraction. These insights are crucial for identifying patients at high risk for timely interventions. Future cohort studies with extended follow-up periods will yield more precise data on causal relationships and the effects of managing these risk factors.

## Figures and Tables

**Table 1:** The baseline characteristics of patients.

Characteristics of Patients	Total (*n* = 579)
Gender	
Male (*n*, %)	223 (38.51%)
Female (*n*, %)	356 (61.49%)
Age (years)	64.04 ± 10.74
BMI	
Underweight, *n* (%)	19 (3.28)
Normal, *n* (%)	153 (26.42)
Overweight, *n* (%)	168 (29.02)
Obesity, *n* (%)	239 (41.28)
Diabetes mellitus, *n* (%)	139 (24.01)
Smoking, *n* (%)	37 (6.39)
Dislipidemia, *n* (%)	96 (16.58)
Control of blood pressure, *n* (%)	258 (44.56)
Duration of hypertension	
≤5 years, *n* (%)	280 (48.35)
6–10 years, *n* (%)	188 (32.48)
>10 years, *n* (%)	111 (19.17)

**Table 2:** The echocardiographic characteristics of patients.

Echocardiographic Characteristics	Total (*n* = 579)
LVEF, %	72.64 ± 4.68
E/A ratio	0.96 ± 0.21
E’ sept m/s	0.08 ± 0.02
E’lat m/s	0.10 ± 0.03
E/E’avg	8.01 ± 2.35
TRV m/s	2.02 ± 0.72
LA volume index (mL/m^2^)	26.68 ± 11.84
LVMI (g/m^2^)	88.48 ± 24.98
RWT	0.32 ± 0.06
GLS (%)	−20.25 ± 2.53

E/A: early to late mitral filling velocity ratio; E/E’: average mitral-to-peak early diastolic annular ratio; GLS: global longitudinal strain; LA: left atrial; LV: left ventricular; RWT: relative wall thickness; LVEF: left ventricular ejection fraction.

**Table 3:** Association between age and echocardiographic measurements.

Echocardiographic Characteristics	Age		
	<60 Year	≥60 Years	*p*
E’ sept m/s	0.09± 0.02	0.07 ± 0.02	<0.001 ^a^
E’lat m/s	0.12 ± 0.03	0.10 ± 0.03	<0.001 ^a^
E/E’avg	7.13 ± 1.67	8.44 ± 2.52	<0.001 ^a^
TRvm/s	0.13 ± 0.02	0.13 ± 0.02	0.339 ^a^
LA volume index	17.3 ± 6.8	18.1 ± 7.2	<0.001 ^a^
LVMI (g/m^2^)	86.44 ± 21.25	89.49 ± 26.59	0.213 ^a^
RWT	0.32 ± 0.05	0.32 ± 0.06	0.803 ^a^
GLS	−20.35 ± 2.58	−20.19 ± 2.50	0.485 ^b^

^a^: Mann–Whitney test; ^b^: *t* test. E’ sept: peak early diastolic tissue velocity at medial mitral annulus; E’ lat: peak early diastolic tissue velocity at lateral mitral annulus; E/A: early to late mitral filling velocity ratio; E/E’avg: average mitral-to-peak early diastolic annular ratio; GLS: global longitudinal strain; LA: left atrial; LVMI: left ventricular mass index; RWT: relative wall thickness; TRv: peak tricuspid regurgitant velocity.

**Table 4:** Association between BMI and echocardiographic measurements.

Echocardiographic Characteristics	BMI			
	Underweight	Normal	Overweight and Obesity	*p*-Value
E’ sept m/s	0.08 ± 0.02	0.08 ± 0.02	0.08 ± 0.02	0.622 ^c^
E’lat m/s	0.10 ± 0.03	0.10 ± 0.03	0.10 ± 0.03	0.607 ^c^
E/E’avg	9.09 ± 4.29	7.92 ± 2.41	7.87 ± 2.37	0.500 ^c^
TRvm/s	0.14 ± 0.02	0.13 ± 0.02	0.13 ± 0.02	0.221 ^c^
LA volume index	16.7 ± 7.3	16.9 ± 7.5	17.5 ± 6.9	0.002 ^a^
LVMI (g/m^2^)	82.87 ± 24.28	85.68 ± 27.84	90.19 ± 23.25	0.015 ^c^
RWT	0.31 ± 0.08	0.31 ± 0.06	0.31 ± 0.05	<0.001 ^a^
GLS	−20.5 ± 3.07	−20.72 ± 2.55	−20.45 ± 2.34	0.002 ^d^

^a^: Mann–Whitney test; ^c^: Kruskal–Wallis test; ^d^: ANOVA one way; E’ sept: peak early diastolic tissue velocity at medial mitral annulus; E’ lat: peak early diastolic tissue velocity at lateral mitral annulus; E/A: early to late mitral filling velocity ratio; E/E’avg: average mitral-to-peak early diastolic annular ratio; GLS: global longitudinal strain; LA: left atrial; LVMI: left ventricular mass index; RWT: relative wall thickness; TRv: peak tricuspid regurgitant velocity.

**Table 5:** Association between diabetes mellitus and echocardiographic measurements.

Echocardiographic Characteristics	Diabetes Mellitus		
	Yes	No	*p*
E’ sept m/s	0.08 ± 0.02	0.07 ± 0.02	<0.001 ^a^
E’lat m/s	0.11 ± 0.03	0.10 ± 0.03	0.002 ^a^
E/E’avg	7.93 ± 2.31	8.26 ± 2.48	0.308 ^a^
TRvm/s	0.13 ± 0.02	0.13 ± 0.02	0.883 ^a^
LA volume index	17.0 ± 7.6	18.4 ± 7.1	<0.001 ^a^
LVMI (g/m^2^)	86.61 ± 23.70	94.42 ± 27.92	0.002 ^a^
RWT	0.31 ± 0.06	0.33 ± 0.07	0.003 ^a^
GLS	−20.49 ± 2.46	−19.48 ± 2.60	<0.001 ^b^

^a^: Mann–Whitney test; ^b^: *t* test. E’ sept: peak early diastolic tissue velocity at medial mitral annulus; E’ lat: peak early diastolic tissue velocity at lateral mitral annulus; E/A: early to late mitral filling velocity ratio; E/E’avg: average mitral-to-peak early diastolic annular ratio; GLS: global longitudinal strain; LA: left atrial; LVMI: left ventricular mass index; RWT: relative wall thickness; TRv: peak tricuspid regurgitant velocity.

**Table 6:** Association between smoking and echocardiographic measurements.

Echocardiographic Characteristics	Smoking		
	Yes	No	*p*
E’ sept m/s	0.08 ± 0.02	0.08 ± 0.02	0.590 ^a^
E’lat m/s	0.10 ± 0.03	0.10 ± 0.02	0.371 ^a^
E/E’avg	8.04 ± 2.39	7.57 ± 1.79	0.283 ^a^
TRvm/s	0.14 ± 0.02	0.13 ± 0.02	0.160 ^a^
LA volume index	17.0 ± 7.3	17.2 ± 7.5	0.382 ^a^
LVMI (g/m^2^)	88.03 ± 24.60	95.22 ± 29.57	0.014 ^a^
RWT	0.31 ± 0.06	0.35 ± 0.08	<0.001 ^a^
GLS	−20.28 ± 2.55	−19.73 ± 2.04	0.199 ^b^

^a^: Mann–Whitney test; ^b^: *t* test. E’ sept: peak early diastolic tissue velocity at medial mitral annulus; E’ lat: peak early diastolic tissue velocity at lateral mitral annulus; E/A: early to late mitral filling velocity ratio; E/E’avg: average mitral-to-peak early diastolic annular ratio; GLS: global longitudinal strain; LA: left atrial; LVMI: left ventricular mass index; RWT: relative wall thickness; TRv: peak tricuspid regurgitant velocity.

**Table 7:** Association between dyslipidemia and echocardiographic measurements.

Echocardiographic Characteristics	Dyslipidemia		
	Yes	No	*p*
E’ sept m/s	0.08 ± 0.02	0.08 ± 0.02	0.019 ^a^
E’lat m/s	0.10 ± 0.03	0.10 ± 0.02	0.336 ^a^
E/E’avg	8.07 ± 2.40	7.70 ± 2.08	0.338 ^a^
TRvm/s	0.13 ± 0.02	0.1 ± 0.03	0.353 ^a^
LA volume index	17.1 ± 7.5	16.9 ± 7.3	0.436 ^a^
LVMI (g/m^2^)	88.32 ± 25.93	89.31 ± 19.57	0.110 ^a^
RWT	0.32 ± 0.06	0.32 ± 0.05	0.044 ^a^
GLS	−20.39 ± 2.43	−19.52 ± 2.87	0.006 ^e^

^a^: Mann–Whitney test; ^e^: Welch’s *t*-test. E’ sept: peak early diastolic tissue velocity at medial mitral annulus; E’ lat: peak early diastolic tissue velocity at lateral mitral annulus; E/A: early to late mitral filling velocity ratio; E/E’avg: average mitral-to-peak early diastolic annular ratio; GLS: global longitudinal strain; LA: left atrial; LVMI: left ventricular mass index; RWT: relative wall thickness; TRv: peak tricuspid regurgitant velocity.

**Table 8:** Association between risk factors and LV diastolic dysfunction.

	LV Diastolic Function		*p*-Value
	Normal (*N* = 317, 54,74%) *N*, %	Abnormal (*N* = 262, 45,25%) *N*, %	
Age			
<60 years	153 (48.26)	38 (14.50)	<0.001 ^f^
≥60 years	164 (51.74)	224 (85.50)	
BMI			
Underweight, *n* (%)	9 (2.84)	10 (3.82)	0.236 ^f^
Normal, *n* (%)	94 (29.65)	59 (22.52)	
Overweight and obesity, *n* (%)	214 (67.51)	193 (73.66)	
Diabetes mellitus, *n* (%)	59 (18.61)	80 (30.53)	0.001 ^f^
Smoking	18 (5.68)	19 (7.25)	0.441 ^g^
Dyslipidemia, *n* (%)	47 (14.83)	49 (18.70)	0.212 ^f^
Duration of hypertension, years	5.08 ± 4.32	5.79 ± 4.58	0.782 ^a^
High blood pressure control, *n* (%)	190 (59.93)	90 (34.35)	<0.001 ^f^

^a^: *t* test, ^f^: Chi-squared test, ^g^: Fisher’s exact test

**Table 9:** Association between risk factors and decrease in GLS.

	GLS		*p*-Value
	Normal (*N* = 526, 90.85%) *N*, %	Abnormal (*N* = 53, 9.15%) *N*, %	
Gender			
Female	328 (62.36)	28 (52.83)	0.174 ^f^
Male	198 (37.64)	25 (47.17)	
Age			
<60 years	171 (32.51)	15 (37.74)	0.034 ^f^
≥60 years	355 (67.49)	38 (62.26)	
BMI			
Underweight, *n* (%)	16 (3.04)	3 (5.66)	0.278 ^f^
Normal, *n* (%)	142 (27.00)	11 (20.75)	
Overweight and obesity, *n* (%)	368 (69.96)	39 (73.58)	
Diabetes mellitus, *n* (%)	117 (22.24)	22 (41.51)	0.002 ^f^
Smoking	35 (6.65)	2 (3.77)	0.564 ^g^
Dyslipidemia, *n* (%)	81 (15.40)	15 (28.30)	0.016 ^f^
Duration of hypertension, years	5.08 ± 3.56	5.79 ± 4.43	0.656 ^a^
High blood pressure control, *n* (%)	252 (98.43)	6 (11.32)	<0.001 ^f^
Abnormalities of diastolic function	225 (42.78)	37 (69.81)	<0.001 ^f^
Type of remodeling			
Normal	420 (79.85)	33 (62.26)	<0.001 ^g^
Concentric LVH	9 (1.71)	6 (11.32)	
Eccentric LVH	87 (16.54)	10 (18.87)	
Concentric remodeling	10 (1.90)	4 (7.55)	

^a^: *t* test, ^f^: Chi-squared test, ^g^: Fisher’s exact test. LVH: left ventricular hypertrophy; GLS: global longitudinal strain.

**Table 10:** Independent factors associated with LV diastolic function by logistic regression.

	OR	95% CI	*p*-Value
Age ≥ 60 years	1.70	1.29–2.12	<0.001
Underweight	1.61	0.59–4.40	0.349
Overweight	1.55	0.96–2.51	0.072
Obesity	1.73	1.11–2.70	0.016
Diabetes mellitus	1.54	1.02–2.33	0.041
Dyslipidemia	1.33	0.82–2.15	0.242
Duration of hypertension	1.02	0.99–1.07	0.734
Uncontrolled blood pressure	1.92	1.77–2.85	<0.001

OR: odds ratio; CI: confidence interval.

**Table 11:** Independent factors associated with abnormal GLS in hypertensive patients by logistic regression.

	OR	95% CI	*p*-Value
Female	1.34	0.71–2.55	0.370
Age ≥ 60 years	2.32	1.67–4.88	0.020
underweight	1.57	0.32–7.69	0.581
Overweight	0.84	0.35–2.05	0.705
Obesity	1.23	0.57–2.70	0.596
Diabetes mellitus	2.06	1.09–3.88	0.026
Smoking	0.38	0.08–1.79	0.218
Dyslipidemia	1.95	0.97–3.91	0.060
Duration of hypertension	0.92	0.88–1.36	0.476
Uncontrolled blood pressure	2.02	1.75–3.99	0.031
LV diastolic dysfunction	3.51	1.74–7.09	<0.001
Concentric LVH	5.78	1.75–19.12	0.004
Eccentric LVH	1.36	0.61–3.01	0.454
Concentric remodeling	4.82	1.34–17.34	0.016

OR: odds ratio; CI: confidence interval; LVH: left ventricular hypertrophy; LV: left ventricular.

## Data Availability

The data presented in this study are available upon request from the corresponding author. The data are not publicly available due to privacy reasons.
